# The evolution of strategies to minimise the risk of human drug-induced liver injury (DILI) in drug discovery and development

**DOI:** 10.1007/s00204-020-02763-w

**Published:** 2020-05-06

**Authors:** Paul A. Walker, Stephanie Ryder, Andrea Lavado, Clive Dilworth, Robert J. Riley

**Affiliations:** 1 Cyprotex Discovery Ltd., No.24 Mereside, Alderley Park, Macclesfield, Cheshire, SK10 4TG UK; 2 Present Address: Alderley Park Accelerator, Alderley Park, Macclesfield, Cheshire, SK10 4TG UK

**Keywords:** Hepatotoxicity, Spheroid, HCI, DILI, *C*_max.tot_, *C*_max,u_, Strategies

## Abstract

Early identification of toxicity associated with new chemical entities (NCEs) is critical in preventing late-stage drug development attrition. Liver injury remains a leading cause of drug failures in clinical trials and post-approval withdrawals reflecting the poor translation between traditional preclinical animal models and human clinical outcomes. For this reason, preclinical strategies have evolved over recent years to incorporate more sophisticated human in vitro cell-based models with multi-parametric endpoints. This review aims to highlight the evolution of the strategies adopted to improve human hepatotoxicity prediction in drug discovery and compares/contrasts these with recent activities in our lab. The key role of human exposure and hepatic drug uptake transporters (e.g. OATPs, OAT2) is also elaborated.

**Electronic supplementary material:**

The online version of this article (10.1007/s00204-020-02763-w) contains supplementary material, which is available to authorized users.

## Introduction

Drug-induced liver injury (DILI) remains a leading cause of drug failure in terms of clinical trials and drug withdrawals (Watkins 2011; Cook et al. 2014). Approximately 18% of compound withdrawals from the market between 1953 and 2013 were due to hepatotoxicity, making the liver the most frequent site of adverse drug reactions (ADRs) leading to drug failure (Onakpoya et al. 2016). Significant inter-species differences in drug absorption, distribution, metabolism, and excretion (ADME), resulting in differences in metabolic fates and the exposure of test compounds in blood and key tissues (Martignoni et al. 2006), confound the extrapolation of data derived from pre-clinical species. Moreover, a large-scale comparison of animal versus human toxicity associated with 150 compounds found that rodent (primarily rat) and non-rodent (primarily dog) animal models predicted only approximately 50% of the human DILI events attributed to these drugs (Olson et al. 2000). More recent analyses have gone so far as to propose that the quality of preclinical safety profiles may actually be inversely correlated with clinical stage project closure due to safety issues (Cook et al. 2014). The pharmaceutical industry has responded to this challenge in the drive to develop human-focused predictive in vitro assay systems to address the risk of hepatotoxicity early in drug discovery. This is evident by the large number of publications (> 100; PubMed) over the last 5–10 years in this field.

In vitro human-based models for the prediction of hepatotoxicity have been developed utilising a range of cell sources and endpoints. These include the use of cell lines, e.g. HepG2, THLE and HepaRG cells and primary human hepatocytes with endpoints ranging from biochemical measurements of (cell death) markers such as LDH leakage or ATP measurement to more mechanistic (pre-cytotoxic) multiplexed endpoint measurement using techniques such as high-content imaging (HCI), as hepatotoxicity involves multiple mechanisms (see below; Lee 2003), and as such a multi-parametric approach may be important for the accurate evaluation of hepatotoxic potential. The current understanding of DILI remains sub-optimal, with a number of marketed drugs (e.g. paracetamol) demonstrating dose-dependent predictable hepatotoxicity, which can often be identified in preclinical animal toxicology studies. Various underlying molecular/cellular mechanisms have been identified as arising from either direct or off-target effects of either the parent drug or its metabolites. However, a substantial number of marketed drugs are idiosyncratic in nature occurring infrequently (typically 1 in 10,000 patients or less) and as such are often only identified following regulatory approval, leading to costly drug withdrawals (Lee 2003). Idiosyncratic DILI is almost undetectable in preclinical animal experiments (Funk and Roth 2017; Robles-Diaz et al. 2016) and therefore poses a significant problem for the pharmaceutical industry. It is increasingly appreciated that delayed, idiosyncratic hepatotoxicity is frequently the result of an adaptive immune attack on the liver (Mosedale and Watkins 2017). However, there are several in vitro studies using multi-parametric approaches shown to identify hepatotoxicants considered idiosyncratic (Aleo et al. 2014; Schadt et al. 2015; Shah et al. 2015; Thompson et al. 2012). These studies propose that many examples of idiosyncratic drug-induced DILI have underlying intrinsic mechanisms that pose a risk for hepatotoxicity. In addition, recent developments have seen a shift in emphasis by the industry from single-cell 2D in vitro approaches to more complex 3D assays and the potential of multicellular microphysiological devices is also being evaluated within a vision to replicate the characteristics and response of human tissues in vivo. This article summarises the evolution of strategies within and across pharmaceutical companies to identify and minimise human DILI liabilities in the drug discovery and development process and recent developments from our own lab.

## Human DILI classification

**Fig. 1:**
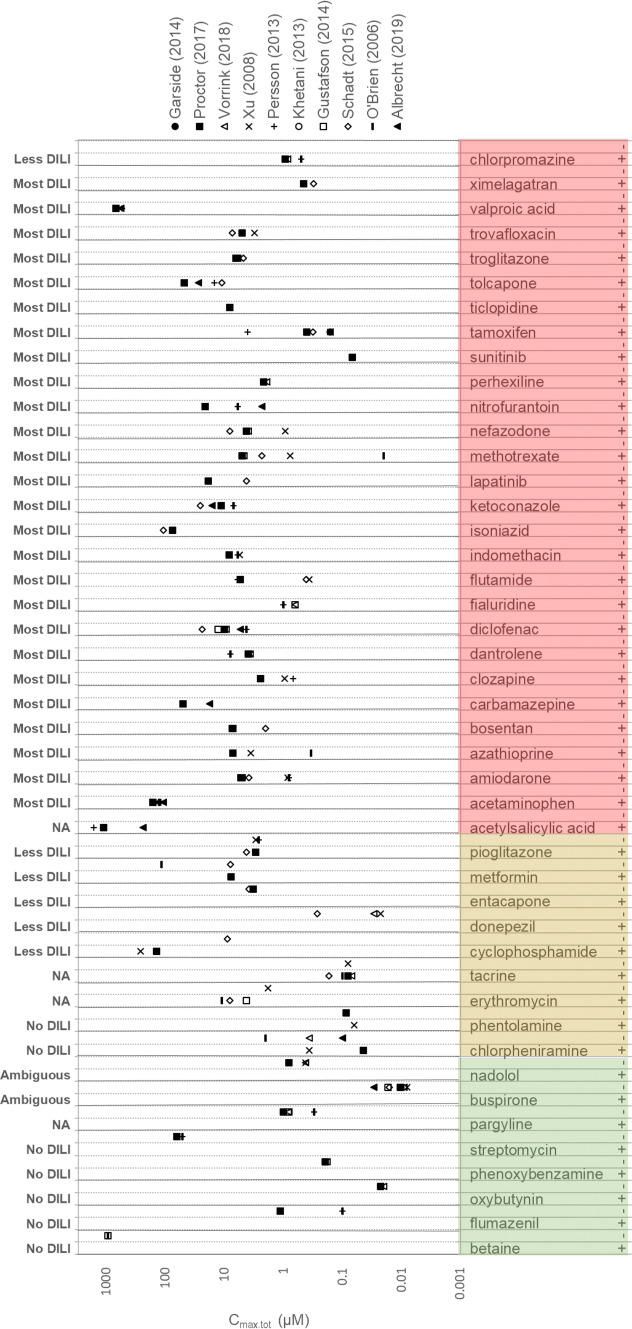
Distribution of assigned DILI categories and *C*_max.tot_ values and across 10 literature references for 33 compounds. *C*_max.tot_ (µM) values are plotted on a log scale when available in the literature. Green shading highlights compounds with negative DILI potential concordance, yellow shading highlights compounds of ambiguous DILI categorisation, red shading highlights compounds with positive DILI potential concordance across the literature. In vivo DILI potential ( +) and no DILI potential (−) are assigned from the literature, aligned with the DILI severity category (top; Chen et al. 2016), unless not available *NA* (color figure online)

First, it is challenging to evaluate and cross-compare directly the relative sensitivity and specificity of DILI strategies from existing publications. This is related to a lack of consistent reference compounds and also inconsistency in the assignment of in vivo DILI categories. Figure 1 highlights inconsistent DILI categorisation with 11 compounds having both positive and negative DILI assignments across the literature. Pioglitazone, for example, is categorised as a Less DILI severity by Chen et al. (2016) but as an in vivo DILI positive by Garside et al. (2014), Proctor et al. (2017) and Gustafsson et al. (2014), and yet O’Brien et al. (2006), Xu et al. (2008), Dawson et al. (2012) and Persson et al. (2013) assume non-DILI potential. To evaluate DILI prediction strategies within early-stage drug discovery and development, our reference compound training sets require standardisation. In an attempt to minimise the discrepancies seen within literature DILI classifications, Chen et al. (2011) published a reference database of drugs (a DILI rank dataset; 287 drugs), which was part of the FDA’s Liver Toxicity Knowledge Base (LTKB) project. This was subsequently followed in 2016 with a larger reference set including 1036 marketed drugs categorised using their developed schema to verify FDA drug label information using publicly available resources such as NIH LiverTox, Spanish DILI registry, Swedish Adverse Drug Reaction Advisory Committee Database and the Drug-Induced Liver Injury Network (DILIN) in the USA (Chen et al. 2016). This DILI classification has started to be incorporated in to more recent studies (e.g. Aleo et al. 2019 and Williams et al. 2019). A comparison of 17 publications was performed over 769 tested reference compounds, aligned with (Chen et al. 2016), where possible (supplemental Table 1).

Supplemental Table 1 contains 1383 DILI and non-DILI reference compounds utilised from 19 publications (Aleo et al. 2019; Albrecht et al. 2019; Chen et al. 2011, 2016; Dawson et al. 2012; Garside et al. 2014; Gustafsson et al. 2014; Khetani et al. 2012; O’Brien et al. 2006; Porceddu et al. 2012; Proctor et al. 2017; Sakatis et al. 2012; Schadt et al. 2015; Tolosa et al. 2012, 2019; Williams et al. 2019). If available and/or interpretable the in vivo DILI observation has been simplified to positive (+ ve) or negative (−ve), and the assay or strategy prediction included, 422 compounds are classified in the LTKB, however, only 189 of these compounds are consistently classified as either DILI negative or positive across the publications. Interestingly 28 compounds are consistent within the publications but disagree with the LTKB classification for example donepezil which has a “less-DILI concern” LTKB classification; however, in five publications donepezil is considered non-DILI. 11 compounds in publications are considered DILI positive in vivo and are “ambiguous DILI concern” or non-DILI in Chen et al. (2016), e.g. furosemide, tolbutamide and trifluoperazine. The Cyprotex reference set of compounds comprised 54 compounds: 29 assigned as hepatotoxic (Most DILI severity), 9 assigned as hepatotoxic (Less DILI severity) and 16 assigned as non-hepatotoxic (No/ambiguous DILI severity) according to Chen et al. (2016) when available; otherwise, a literature average was adopted.

The question remains how many reference DILI compounds would be sufficient to evaluate the different published DILI strategies. To date the greatest number of reference compounds tested in a single publication is the human hepatocyte imaging assay technology (HIAT; Xu et al. 2008, see Table 1). It certainly would be extremely ambitious to test all 1383 reference compounds in Supplemental Table 1. Also, many strategies report high sensitivity and specificity although with less reference compounds. Perhaps an in vitro DILI strategy development consortium could recommend a “gold standard” reference compound set to be adopted with consideration of currently under-predicted reference compounds (see Supplemental Table 1) and consideration of chemical space, therapeutic areas, and therapeutic dose.

**Table 1:** A summary of key information from DILI strategies or assays published from 22 publication

Parameters	O'Brien et al. (2006)	Xu et al. (2008)	Dawson et al. (2012)	Tolosa et al. (2012)	Thompson et al. (2012)	Gustafson et al. (2014)	Sakatis et al. (2012)	Persson et al. (2013)	Garside et al. (2014)	Atienzar et al. (2014)	Tomida et al. (2015)
S, MF, MA	MA	MF	S	MF	MA	S	MF	MF	MA	MA	MA
No. of compounds	243	344	85	78	36	104	223	102	144	51	32
In vivo toxic	146	200	64	66	27	83	113	66	108	40	17
In vivo non-toxic	95	144	21	12	9	21	110	34	36	11	15
Raw data available	Yes	Yes	Yes	No	Yes	Yes	No	Yes	Yes	Yes	No
Longest assay incubation time (hrs)	72	24	0.08	24	24	24	1.5	72	48	168	24
Location of data	TEXT	SUPP	TEXT	NA	TEXT	TEXT	NA	TEXT	SUPP	TEXT	NA
Toxicological descriptor	IC_50_	Fold change	IC_50_	NA	IC_50_, EC_50_	EC_50_	NA	LEC, IC_50_, AC_50_	IC_50_, EC_50_	LC_50_, TI	Toxicity Score
C_max_ available	Yes	Yes	Yes	Yes	No	Yes	No	Yes	Yes	Yes	Yes
Cut off utilised	Yes	Yes	Yes	Yes	Yes	Yes	Yes	Yes	Yes	Yes	Yes
Toxicity defined (Pos/Neg)	Yes	Yes	No	Yes	Yes	No	No	No	Yes	Yes	Yes
Sensitivity and/or Specificity available	Yes	No	No	Yes	Yes	No	No	SENS	Yes	Yes	Yes
Sensitivity	90%*	*51%*	*44%*	94%	100%	22%*	*53%*	64%*	58%	78%	67%
Specificity	98%*	*100%*	*76%*	92%	78%	95%	*86%*	*91%*	75%	73%	73%
DILI score/ category defined	Yes	Yes	Yes	Yes	Yes	Yes	Yes	Yes	Yes	Yes	Yes

Parameters	Shah et al. (2015)	Schadt et al. (2015)	Saito et al. (2016)	Aleo et al. (2019)	Khetani et al. (2012)	Proctor et al. (2017)	Vorrink et al. (2018)	Williams et al. (2019)	Porceddu et al. (2012)	Albretcht et al. (2019)	Tolosa et al. (2019)
S, MF, MA	MA	MA	MA	MA	MA	S	S	MA	MA	S	MA
No. of compounds	125	81	28	200	45	110	123	96	124	30	15
In vivo toxic	70	38	11	79	35	69	70	63	87	14	11
In vivo non-toxic	55	43	17	121	10	41	53	33	37	16	4
Raw data available	No	Yes	Yes	Yes	No	Yes	No	Yes	Yes	Yes	No
Longest assay incubation time (hrs)	48	48	24	72	336	336	36	96	0.5	48	504
Location of data	NA	TEXT	SUPP	SUPP	NA	SUPP	NA	SUPP	TEXT	SUPP	NA
Toxicological descriptor	IC_50_,	IC_50_, TC_50_	LOAEC	IC_50_	TC_50_	IC_50_	NA	EC_50_	EC_20_	EC_10_, EC_50_	NA
C_max_ available	Yes	Yes	Yes	Yes	Yes	Yes	Yes	Yes	Yes	Yes	Yes
Cut off utilised	Yes	Yes	Yes	Yes	Yes	Yes	Yes	Yes	Yes	Yes	Yes
Toxicity defined (Pos/Neg)	Yes	Yes	Yes	No	Yes	Yes	Yes	Yes	Yes	No	No
Sensitivity and/or Specificity available	Yes	Yes	Yes	Yes	Yes	Yes	Yes	Yes	Yes	Yes	No
Sensitivity	33%	76%	100%	70%*	65.7%*	60.9%	69%	87%	94%*	100%	100%*
Specificity	96%	82%	71%	69%*	90%*	85.4%	100%	85%	64%*	88%	100%*
DILI score/ category defined	Yes	Yes	Yes	Yes	Yes	Yes	Yes	Yes	Yes	Yes	Yes

Italic calculated from available published data

*NA* not applicable or not available, *S* single endpoint assay, *MF* multiple features assessed via HCI, *MA* multiple endpoint assays combined in a single strategy

*Highest reported sensitivity and specificity were applicable

### Development and evolution of pre-clinical pharmaceutical DILI strategies

This review is primarily focused on strategies published by the pharmaceutical industry. However, additional publications deemed to have contributed significantly to this area have been included (see Table 1 for an overview of the strategies discussed below).

Screening for hepatotoxicity has been part of pharmaceutical companies’ strategies for several decades. However, most of the initial work in this area focused primarily on the assessment in rat hepatocytes, due to the limited availability of quality human tissue, with measurement of single biochemical endpoints or markers of cell death (e.g. LDH release, MTT, cellular ATP). With the availability of human-based cell lines (e.g. HepG2, THLE and HepaRG) and the improved access and functionality of cryopreserved human hepatocytes, the industry has responded by developing more human-focused models. In addition, it has generally been accepted that, due to the complex nature of human DILI, the measurement of multiple mechanistic cell health endpoints or markers in these systems should hopefully better inform and improve DILI prediction.

Hepatic mechanisms of chemical injury can be quite diverse as illustrated in an initial review by Lee (2003) highlighting several mechanisms of action including calcium homeostasis, steatosis, cholestasis, covalent binding, immune involvement, apoptosis and mitochondrial affects. Following on from this review, Pfizer in 2004 considered the importance of in vitro mechanistic endpoint assays and the potential to predict DILI liabilities for the pharmaceutical industry (Xu et al. 2004). This review compared simple cytotoxicity assays in HepG2 cells to a panel of pre-lethal mechanistic assays covering a range of toxicities: steatosis, cholestasis, phospholipidosis, reactive metabolites, mitochondrial toxicity, oxidative stress and drug interactions. The authors hypothesised that the rational use of one or more of these pre-lethal cellular mechanistic assays in metabolically competent cells may allow scientists to select new candidates with an improved hepatic safety profile. This was the first recommendation that multiplexed screening for drug safety may be beneficial in pharmaceutical strategies. Two leading papers using HCI in either HepG2 cells or primary human hepatocytes (O’Brien et al. 2006; Xu et al. 2008) followed this review.

The O’Brien et al. (2006) paper used HepG2 cells and HCI to screen for drug-induced human hepatotoxicity (see Table 1 for assay details such as number of compounds tested). Reference compounds were assessed with an exposure time of 72 h. After exposure to test compound, the endpoints measured were cell count, nuclear area, mitochondrial membrane potential (MMP), intracellular calcium levels and cell membrane permeability. The HCI approach was developed due to an initial evaluation of historical data from seven individual in vitro cytotoxicity assays (DNA synthesis, protein synthesis, glutathione (GSH) depletion, superoxide induction, caspase-3 induction, membrane integrity and cell viability), each assay alone yielded < 19% DILI reference compound sensitivity. Combination of three of the assays, DNA synthesis, GSH and cell viability improved the sensitivity, however only achieving 25% with a specificity of 83% (Xu et al. 2004; O’Brien et al. 2006). The HCI-based assay dramatically improved sensitivity and specificity (Table 1) with DILI positive defined as the lowest HCI endpoint IC_50_ (or in the absence of dose–response < 100 µM) < 30 × total human plasma exposure levels (*C*_max.tot_). The incorporation of human exposure affords the opportunity to translate an in vitro flag or hazard into a quantitative assessment of risk. The most sensitive features measured were cell proliferation, mitochondria and nuclear area with the least sensitive parameters being membrane permeability and calcium levels. The authors stated these multiplexed models might provide a valuable mechanistic understanding for compound prioritisation or de-risking. However, there are limitations with this approach in particular the lack of metabolic competence in HepG2 cells, previously highlighted by Xu et al. (2004).

Pfizer published subsequently in 2008 on a further HCI approach, the HIAT assay, to predict clinical drug-induced liver injury (Xu et al. 2008). 110 reference compounds overlapped with the previous study with DILI compounds classified as withdrawn from the market due to hepatotoxicity; not marketed in the USA due to hepatotoxicity; assigned black box warnings from the Food and Drug Administration (FDA); marketed with hepatotoxicity warnings independent of clinical reports of hepatotoxicity; and internal Pfizer compounds for which development was stopped due to hepatotoxicity. Any compound not meeting any of the above criteria was assigned as DILI negative. The compounds were assessed in metabolically competent human hepatocytes with a 24-h exposure and concentrations up to 100-fold the total plasma therapeutic *C*_max.tot_ or 1 µM if *C*_max_ values were not available (i.e. top concentration tested was 100 µM). The rationale for the 100-fold ratio was to account for several factors: population exposure variability (×6); potential for higher liver exposure via the portal vein for orally administered drugs (×6); and a further threefold to account for drug–drug and/or drug–diet interactions and potential for accumulation on repeat dosing (Xu et al 2008). The multiplexed endpoints measured were nuclear count, nuclear area, lipid intensity, reactive oxygen species (ROS) formation, MMP and GSH levels. Analysis of the endpoints produced a true-positive rate of 50–60% with a false-positive rate of 0–5% (see Table 1). Analysis of the contribution of individual features to the models’ predictivity showed that MMP, ROS and GSH contributed most to the prediction of DILI with cell number and nuclear count contributing least. The authors utilised a higher ratio of *C*_max.tot_ (100×), which was purported as a reasonable threshold to differentiate between toxic and non-toxic drugs. Further investigations into drug–drug combinations, long-term exposure and additional mechanistic endpoints were considered as additional factors, which could improve the model further with respect to predicting human DILI.

In 2012, AstraZeneca published a study investigating the role of inhibition of the bile salt efflux transporter (BSEP; ABCB11; Dawson et al. 2012) in cholestatic DILI. They study utilised both human and rat BSEP activity in membrane vehicles. 42 drugs were classified as DILI, which are cholestatic/mixed hepatocellular, 22 as DILI, hepatocellular and the remaining 21 as non-DILI. The authors also considered drug dose and maximum unbound plasma concentration (*C*_max,u_) as additional DILI risk factors. The large distribution in *C*_max,u_ values within DILI categories suggested that C_max,u_ alone is not a primary determinant of liver injury. Using an hBSEP inhibition IC_50_ threshold of < 300 µM assigned 24 out of 42 cholestatic/mixed compounds as positive, with 4 positive hepatocellular compounds and 5 false-positives. Three out of the five false-positives had low drug exposures (*C*_max,u_ < 0.002 µM) with only two from the DILI compounds with *C*_max,u_ < 0.002 µM, and combining both cut-off criteria to determine DILI-positive compounds (*C*_max,u_ > 0.002 µM and IC_50_ < 300 µM). The authors concluded that *C*_max,u_ has the potential to improve the assessment of the DILI risk posed by test compounds which inhibit BSEP in vitro, yet accepted that the presence of false-negatives within their assay highlights the existence of more complex relationships and that cholestatic liver injury is only one contributing mechanism of DILI.

Also during this time, a third paper using a HCI approach was published using HepG2 cells at two time points (3 and 24 h) and exposed to either 100 µM or retested at 1000 µM if no response was observed (Tolosa et al. 2012). The HCI endpoints determined were cell viability, nuclear morphology, MMP, intracellular calcium and oxidative stress, several of which could be linked to in vivo mechanism. The minimum effective concentration (MEC) from the HCI data was used and based on the strength of response a toxicity risk (TR) was calculated. Despite a quite high cut-off of 1000 µM high sensitivity and specificity (1 false-positive; ketotifen) was obtained (Table 1). Interestingly, this approach without *C*_max_ normalisation was capable of generating a severity score aligned with DILI in vivo, with this in mind it would be interesting to test a greater number of non-DILI compounds. To address the low metabolic activity in HepG2 cells, the authors later evaluated a small number of bioactivated compounds in HepG2 cells transfected with CYP enzymes (Tolosa et al. 2013, 2018).

In the same year, AstraZeneca also published an internal strategy to screen and mitigate the risk of DILI in drug discovery and development (Thompson et al. 2012). A panel of five in vitro assays was used to assess reference compounds, 16 of which resulted in either discontinued development, withdrawal or FDA black box warnings categorised as severe, 11 resulting in restricted drug usage and cautionary warnings categorised as marked concern, and 9 categorised as low concern which were associated with few findings.

The individual assays utilised were

- Toxicity to THLE cells (SV40 T-antigen-immortalised human liver epithelial cells) which do not express CYP enzymes
- Toxicity to THLE cells which selectively express CYP3A4
- Cytotoxicity in HepG2 cells grown in either glucose or galactose media to determine mitochondrial toxicity
- Inhibition of the human bile salt efflux transporter (BSEP; ABCB11)
- Inhibition of the rat multidrug resistance-associated protein 2 (MRP2)
- Determination of reactive metabolite exposure by determining the covalent binding in human hepatocytes together with the maximum prescribed dose and fraction of metabolism contributing to covalent binding.

Even though drugs classified as severe or marked exhibited activity in individual assays, it was apparent that many examples from these categories did not exhibit any activity (13 out of 27). Furthermore, three out of the low concern drugs responded in at least one assay, therefore a combined endpoint approach was assessed to determine an aggregate score for compounds with data from the individual assays. Using a panel score of two gave sensitivity of 48% and specificity of 89%. The incorporation of dose and covalent binding burden with these panel scores to determine an integrated in vitro hazard matrix, using cut-offs of a panel score > 2 and a CVB burden of > 1 mg/day, improved overall prediction (Table 1). Limitations include a small test set due to the requirement for radiolabelled compound and lack of enzyme activation aside from CYP3A4 as other CYP enzymes bioactivate many drugs. Additional DILI mechanisms could be evaluated by use of multi-parametric HCI approaches as described above. Results support the concept of an integrated approach to risk assessment, which can be utilised in drug discovery to mitigate risk for human ADR’s and was part of AstraZeneca’s strategy at the time of publication.

AstraZeneca continued work in this area by later publishing work utilising the THLE-Null and THLE-3A4 cell models in an automated screening platform within an MTS-reduction assay following 24-h exposure (Gustafsson et al. 2014). The authors utilised literature and US FDA product labels to categorise their compound set into 5 DILI categories; severe, high concern, DILI reports (considered +ve for DILI), ALT elevations and no liver signals (−ve, DILI). Interestingly, considering the perceived importance of CYP mediated metabolism in DILI, Gustafsson et al*.* reported high specificity in both THLE-Null cells (EC_50_ ≤ 200 µM) and THLE-Null/THLE-3A4 EC_50_ ratio (≥ 1.4) yet modest sensitivity (Table 1) with many of the drugs labelled with DILI concerns not responding.

GSK also published an approach in 2012 for the screening of DILI risk in preclinical candidate selection (Sakatis et al. 2012). Either in-house data or literature data were sourced for CYP metabolism-dependent inhibition (MDI, 179 compounds), GSH adduct formation (190 compounds), covalent binding data (53 compounds) and clinical dose for all compounds. A decision tree was developed based upon a response in either CYP MDI (> fivefold cofactor-dependent decrease in IC_50_ of any enzyme) and GSH adduct observed (both cofactor dependent and independent, of any intensity) in conjunction with daily clinical dose estimated to be ≥ 100 mg. If both in vitro assays were negative, the chemical would be progressed to pre-candidate selection. If an in vitro assay is positive addition investigatory GSH assays in S9 or hepatocytes could be performed to confirm the risk. Further, if the clinical dose is also estimated to be ≥ 100 mg termination may be considered, or further consideration of covalent binding can be taken into account; if ≥ 200 pmol equiv/mg protein then the progression of the compound would be reviewed. 76% of drugs with a daily dose < 100 mg were non-hepatotoxic from the authors’ analysis, ~ 65% of hepatotoxic drugs presented with ≥ 100 mg daily dose or marked GSH adduct formation, marked CYP MDI, or covalent binding ≥ 200 pmol equiv/mg. Unfortunately, the raw data were not provided; however, an analysis of the performance of this approach was calculated (see Table 1 and Supplemental Table 1).

Subsequently, H. Lundbeck A/S reported their approach to predict human drug-induced liver injury (Persson et al. 2013). They developed a novel HCI assay based on the measurement of six parameters: nuclei size, plasma membrane integrity, lysosomal activity, MMP and mitochondrial area in HepG2 cells. HepG2 cells were exposed to the compounds for 24 or 72 h at a fixed concentration range of 0.01–100 µM. Using a 100-fold therapeutic index (TI) which is the ratio of the in vitro MEC and therapeutic *C*_max.tot_ to classify a hepatotoxic and non-hepatotoxic, most individual parameters had a sensitivity and specificity of ~ 50% and ~ 90%, respectively. The authors stated that one of the main priorities of such an early DILI screen should be to avoid deselecting potentially promising compounds in drug discovery programs. Using a zonal classification system based on the nuclei size, MMP and human *C*_max.tot_ values, they were able to identify an area without a single false-positive compound, whilst still maintaining sensitivity (Table 1). This substantiated the initial observation by O’Brien et al. (2006) that nuclei size and MMP are highly predictive parameters for the prediction of human DILI. The lack of sensitivity is again likely due to the low metabolic capability of HepG2 cells (as above), with compounds that require metabolic activation such as bromfenac were negative. The authors concluded that an approach as described above utilised in a drug discovery setting allows for decision making by project teams to prioritise hit series during the hit-to-lead process and allows for the de-risking of potential human DILI liabilities.

AstraZeneca reported an alternative strategy from their earlier combined mechanistic assay approach in 2012 with an HCI DILI strategy in 2014 (Garside et al. 2014). HepG2 cells, HepG2 combined with Aroclor-induced rat liver S9 (rS9) and primary human hepatocytes were used. Cells were exposed to reference compounds (up to 250 µM) and multiplexed endpoints measured at 4 h and 24 h (cell count, MMP, ROS formation), 24 h and 24 h with 24-h recovery (caspase-3 activation), 24 h with 24-h recovery only (cell stress responses; hsp70/72 and cell cycle arrest; pH3) and 48 h (phospholipidosis and neutral lipid accumulation). The individual endpoint that identified DILI with the greatest precision was ROS formation in hepatocytes after 24-h exposure with a sensitivity of 41% and specificity of 86%. The authors refrained from normalising the in vitro data to unbound in vivo plasma concentrations due to concern over assumptions that drugs added to the cells remain free in solution and that there is no binding to proteins or lipids present in the media or cell, plus cellular accumulation related to carrier-mediated transport. The inclusion of rS9 with HepG2 cells did not show any significant improvement in detecting DILI compounds other than cyclophosphamide, a compound known to be metabolised by CYP2C19 and CYP3A4 to a cytotoxic metabolite (Otto et al. 2008; Steinbrecht et al. 2019). However, utilising a hierarchical clustering approach to group drugs based on the similarity of their assay profiles (all in vitro endpoints utilised) provided the most sensitive approach (Table 1). The authors concluded that if this approach were used to support compound profiling in drug discovery it would aid in the selection of compounds with a low risk of DILI liability and potentially other target organ toxicities.

In this same year, UCB published an evaluation of a liver co-culture model (Atienzar et al. 2014). Dog hepatocytes combined with non-parenchymal stromal cells, primary human hepatocytes and HepG2 cells were used and dosed for 5 days with a repeat dose at day 3. Cell viability was determined by measuring cellular protease activity and GSH content with a response (LC_50_) from either assay at less than 100 × *C*_max.tot_ considered hepatotoxic. As such, most of the human hepatotoxic drugs were detected in HepG2 cells, primary human hepatocytes and in the dog co-culture model with a sensitivity of 82, 83 and 78%, respectively. Nevertheless, the specificity was low for HepG2 cells (36%) and 46% for hepatocytes compared to 73% for the canine model; however, only 11 non-hepatoxic compounds were assessed (Table 1). It is also well accepted that primary hepatocytes’ cultures are limited in their ability to maintain hepatocyte functionality over an extended time course (Lecluyse 2001).The overall higher specificity of the liver co-culture models could be a consequence of their ability to mimic better in vivo liver morphology and functions such as albumin and urea production as well as active bile canaliculi.

Tomida et al. (2015) from Kaken Pharmaceuticals used HepaRG cells as a human liver in vitro model following studies showing that HepaRG cells maintain many liver-specific functions such as expression of CYP enzymes, nuclear receptors, membrane transporters, and phase II metabolising enzymes at levels comparable to those of human primary hepatocytes (Aninat et al. 2006; Guillouzo et al. 2007). HepaRG cells were exposed to the test set of compounds for 24 h at a range of concentrations equivalent to 1.6-, 6.3-, 25- and 100-fold the therapeutic *C*_max.tot_. Following treatment cellular health was assessed using several endpoints: cell viability, GSH content, caspase 3/7 activity, lipid accumulation, LDH leakage and albumin secretion. A positive in one endpoint from the multi-parametric assay was determined if cut-off values of < 70%, < 60%, > 4.9-fold, > 2.8-fold, > 1.9-fold and < 40%, respectively, were reached within 100 × *C*_max.tot._ This approach gave a sensitivity of 67% and specificity of 73%. Applying a 25 × *C*_max.tot_ cut-off decreased the sensitivity to 41% with a concurrent increase in specificity to 87%, a relatively low sensitivity compared to other studies (see Table 1).

In 2015, Pfizer also published a strategy to combine hepatotoxic liabilities (cytotoxicity in THLE or HepG2 cells), BSEP inhibition, or mitochondrial inhibition/uncoupling (Shah et al. 2015). A *C*_max.tot_ of > 1.1 µM alone distinguished most DILI from non-DILI compounds with high sensitivity (80%) and specificity (73%), whereas *C*_max.u_ alone gave a sensitivity of 52% and specificity of 74% at > 0.51 µM. The sensitivity of the three assays BSEP, mitochondrial effects and cytotoxicity was relatively low with *C*_max.tot_ > 1 µM and IC_50_ < 100 µM providing 33, 20 and 27% sensitivity respectively with specificity ≥ 96%. A combination of all three approaches gave poor sensitivity of with no false-positives (Table 1).

Roche also published their approach in 2015 (Schadt et al. 2015) which utilised a range of in vitro endpoints to minimise DILI risk in development. The endpoints measured were generation of reactive metabolites (CYP3A4 time-dependent inhibition and GSH-adduct formation), BSEP inhibition, mitochondrial toxicity and cytotoxicity in NIH3T3 fibroblasts and primary human hepatocytes. Compounds were classified according to FDA drug labelling (Chen et al. 2011). A comparison of the predictivity of individual endpoints was calibrated with either dose or plasma exposure from typical clinical dosing regimens. Incorporating all endpoints and calibrating with dose-reported sensitivity and specificity values of 76% and 82%, the specificity is amongst the lowest reported (Table 1) The authors conclude that the best option to mitigate the risk of idiosyncratic DILI potential in drug discovery and development is to reduce the major compound-related risk factors, by utilising mechanistic screening assays with rapid turnaround times to support lead optimisation.

Astellas Pharmaceuticals published an HCI approach for the prediction of human hepatotoxicity (Saito et al. 2016). In this study, the in vitro systems assessed were HepG2 and HepaRG cells cultured as monolayers. The two liver-derived cell lines were exposed to the test compounds for 1, 6 and 24 h after which time the following endpoints were measured: nuclear count, nuclei size and intensity, ROS intensity, membrane permeability, MMP intensity, GSH content and cellular ATP. The lowest observable adverse effect concentration (LOAEC) was determined for each of the parameters, broadly equivalent to an MEC threshold used in other studies. Comparison of the responses in HepG2 cells versus HepaRG cells showed that changes to ROS generation and GSH decreases were observed at lower concentrations in HepaRG compared with HepG2 cells. In addition, seven of these compounds were known to form CYP-dependent GSH conjugates highlighting the metabolic differences between the two cell types. In addition, the authors utilised a scoring approach to predict human DILI potential based upon incorporating all of the individual endpoints. Using an approach based upon two individual parameters responding at 100 × *C*_max.tot_ gave a sensitivity and specificity of 90.9% and 76.6% in HepaRG cells and 100% and 70.6% in HepG2 cells. More compounds would need testing to confirm which cell model is most predictive and also if the high sensitivity observed.

Sison-Young et al. (2017) published on a pharmaceutical multi-centre assessment of single-cell models for prediction of hepatotoxicity. This work was part of the EU-funded MIP-DILI project focused on the development and evaluation of in vitro approaches for the prediction of human DILI. The cell models assessed incorporated primary human hepatocytes, HepG2, Upcyte and HepaRG cells. A relatively small compound set was used to assess the predictivity of the models with nine DILI compounds and four non-DILI compounds screened in each. Cells were dosed for either 24 h or dosed daily over a 72-h period with cell viability determined (cellular ATP). This approach was taken to assess critically the cell models utilised across the pharmaceutical industry, with the results indicating that none of the four cell models assessed could distinguish between DILI and non-DILI compounds based upon the in vitro data alone. The authors concluded that particularly when using simple endpoints none of these models were suitable for the prediction of DILI. However, when EC_50_ < 20 × *C*_max.tot_ was incorporated, primary human hepatocytes were the most accurate model for identifying DILI with eight out of the nine DILI true-positive (TP) compounds detected after 72 h but not 24 h with one false-positive (FP), closely followed by HepG2 cells (7 TP and 0 FP). The recommendation was that more complex cells’ models need to be assessed. These could include co-culture models with the incorporation of non-parenchymal cells or long-term exposure in 3D approaches in conjunction with more sophisticated endpoints for adverse cell responses. This was also acknowledged in a perspective from the pharmaceutical industry IQ Consortium DrugSafe (Butler et al. 2017).

Pfizer recently published their latest DILI strategy using modelling approaches instead of binary endpoint analysis (Aleo et al. 2019). The in vitro assays used were cytotoxicity in THLE or HepG2 cells, mitochondrial dysfunction (inhibition and uncoupling) using isolated mitochondria, plus the Glu/Gal assay and bile salt export pump (BSEP) inhibition, screened against a library of 200 reference drugs from the LTKB annotated as Most (79), Less (56), No (47), and Ambiguous-DILI-concern (18). A safety margin was calculated for each in vitro assay by dividing the IC_50_ (μM) values by a *C*_max.tot_ with the exception of the Glu/Gal assay. Hepatic risk matrix (HRM) scores were assigned to each (< 1, 1–10, 10–100, and > 100 × clinical *C*_max,total_ = 4,3,2,1,0, respectively). The Glu/Gal assay was score in the absence of dose normalisation (> 3, > 2 to < 3 and < 2 ratio scoring 4, 2 and 0, respectively). The total of these scores was combined with either of two scores from physiochemical models, which were Rule of Two (cLogP ≥ 3 and total daily dose ≥ 100 mg; Chen et al. 2013) giving a score of 4 or drug ionisation state partitions for daily dose, cLogP, and fractional carbon bond saturation (Fsp3; Leeson 2018) depending upon if the drug was an acid, base or neutral giving a maximum score of 4. These two approaches were then classified as DILI positive with two cut-offs for each (rule of two hybrid HRM scoring system ≥ 3 or ≥ 8; partition hybrid HRM scoring system ≥ 4 or ≥ 8). This approach attempted to predict most-DILI-concern compounds only and considered Less-DILI as part of the negatives, unlike other authors. The sensitivity and specificity of this modelling approach were 70% and 69% respectively for the ≥ 3 score or 41% and 97% respectively for the ≥ 8 score for the HRM combined with Rule of two systems. For the HRM combined with partition system, the sensitivity and specificity were 80% and 58% respectively for the ≥ 4 score or 48% and 94% respectively for the ≥ 8 score. Interestingly using this approach but classifying Less-DILI compounds as positive would have similar specificity with scores > 7.5; however, the sensitivity would drop by > 10% (Table 1). Furthermore, the incorporating of other mechanistic assays (reactive metabolite and cytotoxic metabolite generation and hepatic efflux transport inhibition, other than BSEP) to the HRM had minimal beneficial impact in DILI prediction. Using the Partition HRM hybrid scoring model successfully classified TAK-875 (liver injury in Phase 3 clinical trials) as a DILI concern and was also valuable in evaluating the relevant DILI risk for several compounds in development (Aleo et al. 2019).

During the drafting of this manuscript, the Horizon 2020 EuToxRisk project published an initial DILI strategy (Albrecht et al. 2019). The authors evaluated cytotoxicity in both PHH (three donors) and HepG2 cells, concluding the PHH approach to be more robust. Using an evaluation of test performance, parameters by quantifying the differentiation of DILI and non-DILI compounds (Toxicity Separation Index; TSI) and the degree to which hepatic blood concentrations in vivo can be estimated (Toxicity Estimation Index; TEI). The TSI and TEI evaluation indicated a 48-h exposure was comparable to 7 days and an improvement over 24 h. This finding is in contrast to previous studies that have demonstrated the improved predictive power of repeat dosing over a longer exposure period (Bell et al. 2016; Proctor et al. 2017). In addition, the best parameter of dose normalisation was the 95%-population-based percentile of *C*_max.tot_ (comparable to previous utilised *C*_max.tot_ values, see Fig. 1), rather than *C*_max.u_ as also reported previously (e.g. Shah et al. 2015). With a support vector machine-based classifier, using an EC_10_ − 1.75 TSI cut-off from the median donor–response gave high accuracy of 93% (two false-positives: acetaminophen at *C*_max.tot_ = 109 µM and glucose). This TSI approach is reported to allow extrapolation of DILI risk by generating the probability of hepatotoxic oral doses and blood concentrations. Interestingly, due to the separation between DILI and non-DILI compounds observed, a *C*_max.tot_ cut-off ranging from < 31× to < 99× would yield equivalent sensitivity and specificity. As such, it would be interesting to see the performance of this approach with a larger dataset.

## Evaluation of 3D approaches for DILI evaluation

Pharmaceutical DILI strategies within the industry have gravitated towards evaluating 3D cellular systems as potentially more predictive models of the DILI response in humans. Butler et al. (2017) stated that the predictivity of 2D cellular models may not be adequate hence the drive to build and evaluate more complex in vitro systems. The concept of 3D cell culture models is not new and 3D models utilising a range of cell types have been in existence for many years. However, improvements in cell supplies and in vitro techniques have allowed this approach to be become more accessible, reproducible, cost-effective and less labour-intensive thereby making it more amenable for use in screening strategies.

Tissue function cannot be readily reproduced in vitro without the appropriate tissue architecture mimicking the in vivo scenario. Concerning the liver, the functional unit for drug metabolism and hepatotoxicity is the hepatic lobule consisting of primarily hepatocytes with Kupffer cells, stellate cells and endothelial cells radiating out from a central vein. The apical membrane of hepatocytes forms the continuous network of bile canaliculi and the basal membrane makes contact with the sinusoidal network. As such, the liver architecture is complicated to recapitulate in vitro ideally requiring a polarised multicellular system, with a bile and sinusoidal network and vasculature (see review by Lelièvre et al. 2017 on 3D in vitro model development). Hepatocytes cultured on fibronectin or collagen sandwiches maintain hepatocyte polarity, express functional transporters in culture and are a useful tool to predict hepatobiliary transport in vivo (Bi et al. 2006; Ziegerer et al. 2016). However, sandwich cultures have been shown to de-differentiate by selective remodelling of the mitochondrial and metabolic proteomes (Rowe et al. 2013; Heslop et al. 2017), with resulting decline in liver functions within 24 h (Khetani and Bhatia 2008).

Following on from the HCI approaches in sandwich cultures (the HIAT assay, Xu et al. 2008), Pfizer published their assessment of a 3D DILI assay amenable to long-term drug dosing (Khetani et al. 2012). They utilised human hepatocyte micropatterned co-cultures (hu-MPCC) in combination with a multi-parametric approach monitoring albumin production, ATP content, urea secretion and GSH content following compound exposure. Reference compounds were tested using hu-MPCC (at 1, 30, 60, and 100 × *C*_max.tot_, with 2 repeat doses over 5 days), the compounds were selected and evaluated against negative DILI compounds in the HIAT. A positive was considered if hu-MPCC gave a TC_50_ in at least one of the multiplexed assays at 100 × *C*_max.tot_. All HIAT-positive compounds were also positive in hu-MPCC, and 12/25 DILI compounds negative in HIAT were positive in hu-MPCC, however, with a gain in one false-positive. As suggested here due to differences in in vivo DILI assignments across studies, alignment with the Liver Toxicity Knowledge Base (LTKB; Chen et al. 2011) should help resolve these cross-lab and system comparisons.

One limitation of the hu-MPCC is that long-term dosing is not possible across various donors due to the decline in liver function in serum-free dosing medium (beyond 9 days; Khetani et al. 2012). To move beyond this potential limitation the pharmaceutical industry has looked towards scaffold-free 3D models either using a hanging drop methodology or utilising ultra-low attachment (ULA) plates (Messner et al. 2013; Proctor et al. 2017; Bell et al. 2016). Bell et al. (2016) reported on the development and characterisation of a 3D primary human hepatocyte (PHH) model system for DILI as part of the MIP-DILI project. Using the ULA method to form 3D hepatocyte spheroids, the authors showed that PHH spheroids can be maintained for at least 5 weeks in serum-free conditions: a considerable increase in the potential to perform long-term dosing whilst minimising the binding of drugs or their metabolites to serum proteins which may impact drug sensitivity. Further characterisation also found that these spheroids maintained albumin secretion and CYP levels over this period. By evaluating the response to five hepatotoxins (amiodarone, bosentan, diclofenac, fialuridine and tolcapone) over a range of time points (48 h, 7 days and 28 days) using three hepatocyte donors, it was observed that repeat long-term dosing improves sensitivity to DILI compounds. The ATP EC_50_ values were lower than 30 × *C*_max.tot_ for all five compounds following a 7-day exposure. This was particularly evident for fialuridine, which showed no toxicity at 48-h exposure with increasing toxic response observed at 7 and 28 days. Based upon these responses, albeit with a limited number of reference compounds indicated, this 3D system could be a powerful tool to predict DILI during preclinical drug development. Interestingly, the inter-donor sensitivity of 3D hepatocytes appeared relatively minor depending upon the cut-off used, despite previous observations of differential liver donor metabolic capacity (Shimada et al. 1994; Takayama et al. 2014).

The previous study was followed by Proctor et al. (2017) who published an approach that again was part of the MIP-DILI project, which included Genentech and AstraZeneca. This approach compared the use of PHH in both 2D and 3D formats. Drugs were assigned into one of five categories with respect to DILI classification (Garside et al. 2014) as opposed to the DILI rank dataset (Chen et al 2016; see Supplemental Table 1). For this study, those drugs assigned severe, high and low clinical DILI concern were all considered DILI positive and those drugs classified by elevations in ALT and other enzymes in the clinic with no DILI clinical observations were considered non-DILI. The approach compared the response of PHH cultured in a collagen-coated 2D format exposed for 48 h with the response in 3D PHH co-cultured with Kupffer cells (human liver microtissues; hLiMTs) using the hanging drop method (Messner et al. 2013), exposed for 14 days with repeat dosing. In both assays, the endpoint used to measure viability was cellular ATP, a range of thresholds was used to determine the predictivity of both approaches. Using a predefined IC_50_ threshold of 100 µM, the sensitivity and specificity of 2D were 33.3% and 85.4%, respectively; this increased to 60.9% and 85.4% in hLiMTs. Interestingly normalising the assay responses to *C*_max.tot_ (IC_50_ < 100 × *C*_max.tot_) slightly improved the sensitivity of 2D cultures increasing to 40.6% with no change in specificity. In direct contrast to previous studies such as Shah et al. (2015) where a *C*_max.tot_ cut-off alone (> 1.3 µM) was able to distinguish DILI from non-DILI (sensitivity 73%, specificity 73%); in this study, *C*_max.tot_ normalisation actually decreased the sensitivity and specificity slightly (to 59.4% and 80.5% respectively). However, not all compounds were tested to 100 × *C*_max.tot_, due to solubility, higher concentrations have been tested in other studies. In addition, the finding from Bell et al. (2016) was confirmed whereby long-term dosing in combination with increased repeat dosing improved the sensitivity of 3D models. The 3D-based approach improved prediction of human DILI when compared to a comparative approach in 2D; however, there were still a significant number of DILI compounds (23) incorrectly classified (~ 40%, where IC_50_ > 100 µM).

To assess the reproducibility, robustness and a matched comparison of PHH 2D sandwich cultures and 3D spheroids formed using ULA plates for predicting DILI, Bell et al. (2018) published a multi-centre approach supported by the MIP-DILI project that included AstraZeneca, Janssen Pharmaceuticals, GSK and Orion Pharma. The approach characterised the proteomic phenotype and drug metabolising capability of primary human hepatocytes in the two cellular models. In addition, five DILI compounds were screened in both models across six participating laboratories, and with hepatocytes from three different donors, viability was assessed by measurement of cellular ATP. Repeat dosing was performed in matched conditions over 72 h (single treatment), 7 days (three treatments) and 14 days (six treatments) in both models. The authors concluded that primary human hepatocytes cultured as 3D spheroids were more functionally stable and exhibited increased sensitivity, following repeat dosing, for the prediction of hepatotoxicity when compared to the responses in 2D culture. They proposed that differences in the proteomic phenotype and metabolic capability of the 3D spheroids may underlie some of the rationale for the increased sensitivity as the dosing regime and time points were matched. 3D spheroids, for example, demonstrated higher CYP1A2, CYP2C8, CYP3A4 and drug transporter activity when compared to levels in PHH 2D sandwich cultures. In addition, the inter-laboratory variability was similar between both models. The authors also concluded there was little significance in sensitivity between 3D spheroids from different donors. Nonetheless, bosentan and diclofenac were only detected by one of three donors at a 10 × *C*_max.tot_ cut-off following a 14-day exposure.

In a further publication from the MIP-DILI project by Vorrink et al. (2018), a 3D PHH spheroid model was studied, in chemically defined media, formed using ULA plates. A repeated drug-dosing regimen (six in total) over 14 days was used with an assessment of cellular ATP (Vorrink et al. 2018). Spheroids were exposed to 1×, 5×, and 20 × *C*_max.tot_ concentrations with 48 of the 70 DILI-associated compounds correctly predicting positive (IC_20_ < 20 × *C*_max.tot_ cut-off) and all 53 of the non-DILI compounds were correctly predicted as negative. Unfortunately, the raw data were not provided (see Table 1), as such it is only possible to ascertain from a colour chart which compounds are positive and negative at each concertation. Two compounds, iproniazid (DILI) and propranolol (non-DILI), are positive at 5 × *C*_max.tot_ but curiously not positive at higher concentrations, the rationale for which is not given. A cross-comparison of human hepatocyte spheroids with those from animals used in pre-clinical studies (mouse, Wistar rat, mini-pig and rhesus monkey) was also investigated using 11 compounds (4 non-DILI and 7 DILI). The human hepatocyte spheroid model displayed the highest degree of correlation with clinical DILI which again emphasised the requirement for human cell-based models. The authors argued that whilst studies in pre-clinical species constitute indispensable regulatory requirements, in vitro 3D human-relevant hepatic models can help to minimise late drug-development failures resulting from inter-species differences. An example of this is the case of fialuridine whereby pre-clinical animal studies failed to predict the resulting fatal hepatotoxicity exhibited during phase I clinical trials (Manning and Swartz 1995; McKenzie et al. 1995) and yet PHH spheroid models combined with chronic compound exposure can detect this hepatotoxicity.

AstraZeneca in 2019 published their latest DILI strategy using a Bayesian machine learning modelling approach (Williams et al. 2019). This strategy is the first approach to combine a 3D liver model (HepG2 C3A spheroid), in vitro assays (BSEP, mitochondrial toxicity; Glu/Gal in HepG2 cells and bioactiviation; BA) with physiochemical parameters (cLogP) and exposure (*C*_max.tot_). The relative contribution of the 3D model is difficult to ascertain due to the Bayesian modelling approach applied, however, using a human exposure normalisation approach (e.g. Vorrink et al. 2018; EC_50_ < 20 × *C*_max.tot_) gives a sensitivity and specificity of 68% and 82%, respectively (see Table 1 for Bayesian approach). As the reference compounds do not overlap entirely with that of Vorrink et al. (2018), it is difficult to directly compare; however, the lower accuracy of this 3D model maybe due to the fact that the C3A clone of HepG2 cells was used rather than PHH. C3A spheroids have considerably lower CYP activity; in addition, only a 4-day time course was utilised. AstraZeneca are reputedly developing a high-throughput PHH spheroid assay, which could be incorporated into this approach. *C*_max.tot_ was reported as a high contributor to the DILI prediction, as reported by Proctor et al. (2017) along with bioactivation. In this study, BA flags were collated from the literature and from the NIH toxicity database in line with the FDA (Chen et al. 2016). The literature data were considered from evidence of covalent binding either in vitro or in vivo or thioether adducts and/or conjugates detected by mass spectrometry analysis. Interestingly, 42 of the reference drugs had a BA flag (67% sensitivity) of which zero were false-positives and five of these were classified as DILI with no other in vitro flag. The Bayesian model has been implemented within AstraZeneca since August 2017 as a compound selection tool, and due to the flexibility of the approach novel/value add in vitro assays can be added or replace existing assays moving forward.

During the processing of this review, the MIP-DILI consortium published a comprehensive synopsis of the applicability of cellular models used in DILI research. The authors proposed a “roadmap” or tiered strategy to de-risk DILI using predictive preclinical models. This strategy is based upon a three-tiered approach following in silico tools with increasing complexity of cellular model from single cell to multicellular 3D models and preclinical or patient-derived models. Cellular models are critically evaluated in terms of their ability to determine drug-associated mitochondrial dysfunction, transporter interaction, reactive metabolites and oxidative stress, endoplasmic reticulum stress and immunological response with relevance to DILI in vivo. The suggested roadmap has not yet been evaluated with a reference set of DILI compounds but does provide a strong rationale for the selection of appropriate endpoints and cellular models for future strategies to consider (Weaver et al. 2020).

### Rational for development of 3D approaches with multi-parametric endpoint measurements to predict DILI

As detailed so far, the pharmaceutical industry has taken a diverse approach to mitigate the risk of human DILI in drug discovery. However, as the previous synopsis has highlighted, there are some emerging trends:

1. The need for consistent assignment of DILI class or category.
2. The use of metabolically competent cells such as primary hepatocytes or HepaRG cells appears to be a key element of many strategies (e.g. Saito et al. 2016; Vorrink et al. 2018).
3. Measurement of multiple mechanistic endpoints can improve sensitivity and allows for the detection of differing mechanisms associated with DILI. This can either be done by multiple individual in vitro assays (e.g. Sakatis et al. 2012, Thompson et al. 2012, Aleo et al. 2019 and Williams et al. 2019) or by utilising high-content imaging (HCI) measuring a number of cell health parameters (e.g. Xu et al. 2008, Garside et al. 2014 and Saito et al. 2016).
4. There is an acknowledgement of donor to donor variability with primary human hepatocytes (Shimada et al. 1994; Takayama et al. 2014); however, to date limited donor comparisons in 3D models have shown significant differences (e.g. Sison-Young et al. 2017 and Bell et al. 2018). As such inter-donor variability warrants further investigation.
5. More recently, significant investigations have been conducted into the utility of 3D hepatocyte cultures, which have been shown to maintain liver phenotype, allowing for longer compound exposure, to predict DILI. Recent advances in cell culture techniques such as the hanging drop method or the use of ULA plates have made the creation of 3D cell culture models much more amenable to screening in drug discovery (Proctor et al. 2017 and Bell et al. 2017).
6. The use of extended exposure with repeated dosing regimens appears to be a key in improving the prediction of human DILI (Bell et al. 2016; Proctor et al. 2017), as it is a basic tenet that toxicity is a function of dose (or exposure) and time: DILI in humans often manifests itself following chronic exposure.
7. In general, incorporation of plasma exposure levels (*C*_max.tot_) with in vitro data improves DILI prediction (a notable exception being Proctor et al 2017), emphasising the importance of early estimates/simulations of human PK and exposure in drug discovery (Yoon et al. 2012). The technical feasibility of testing compounds at multiples of human exposure (e.g. 100 × *C*_max.tot_) should also be considered. For example, a compound which requires an unbound *C*_max_ for efficacy of 300 nM and is 99% bound to plasma proteins would require testing at 3 mM, which will likely be impacted by its solubility.

With these considerations in mind, a multi-parametric HCI approach in 3D liver models is compared to recent publications (see Table 3 and below). Some of these emerging themes and their rationale are expanded in the next section.

## Assay formats and endpoints (lethal or pre-lethal/mechanistic)

As summarised in point 3 the reason that multiplexed approaches have gained such momentum is that it is widely acknowledged that DILI can occur via a number of mechanisms; hence, the industry has responded in developing appropriate test systems in drug discovery.

Given the pathological complexity of human DILI, examples of mechanistic endpoints include

- Reactive metabolite formation/conjugation with GSH
- Mitochondrial dysfunction
- Changes in calcium homeostasis
- Bile transporter interactions/regulation
- Immune-mediated effects
  o Immune activation
  o TNF receptor sensitivity

As discussed within this review, global comparisons of DILI strategies are confounded by limited overlap in compound reference sets and *C*_max.tot_ ambiguity (use of total or unbound and absolute values reported) which becomes further confounded by variable DILI classifications and assay significance cut-offs utilised. In this regard, Table 2 summarises a 54-compound reference set (from 10 publications) simplified in terms of positive (most DILI severity and less DILI severity) or negative (no/ambiguous DILI severity) as classified by Chen et al. (2016). Dawson et al. (2012) and Schadt et al. (2015) used BSEP inhibition or metabolism-based approaches to achieve 39% and 76% accuracy, respectively. The higher predictive power reported by Schadt et al. (2015) is likely the result of the multiplexed assay approach utilised, this has been further developed by utilising modelling and Bayesian machine learning to move beyond binary predictions from multiplexed assays (Aleo et al. 2019 and Williams et al. 2019, accuracy of 59–81% and 90%, respectively). O’Brien et al. (2006), Tolosa et al. (2012) and Xu et al. (2008) all utilised HCI-based approaches and reported similar accuracies (88%, 89%, and 67%, respectively). Interestingly, Xu et al. (2008) utilised primary human hepatocytes in their approach as opposed to HepG2 and ultimately achieved the lowest accuracy perhaps suggesting that data from 2D in vitro cultured primary human hepatocytes are not able to translate fully to in vivo (although compound overlap is poor). Comparison of 3D hepatotoxicity approaches shows differing accuracy across these approaches, however, with the largest overlap of reference compounds [Proctor et al. 2017; 67% (µM cut-off) or 76% (C_max.tot_ cut-off), Vorrink et al. 2018; 93%, HCI-hLiMTs; 91% and HCI-HepaRG spheroid; 93%]. Vorrink et al. (2018) using chemically modified culture conditions for PHH spheroids and a single cytotoxicity endpoint (IC_20_ < 20 × *C*_max.tot_ cut-off) showed improved sensitivity in comparison to hLiMTs also using an ATP endpoint (Proctor et al. 2017; IC_50_ < 100 × *C*_max.tot_ or IC_50_ < 100 µM). The authors mention certain cell culture media may downregulate CYP activity; however, as the media composition was not specified, any difference between the two studies would be speculation. Interestingly, the higher assay accuracy is also confirmed in both HepaRG spheroids and hLiMTs using multiple-HCI and ATP endpoints with defined media. In all 3D approaches, bosentan and sitaxsentan were both positive but not in PHH assays. Both compounds have been implicated in BSEP transport inhibition and mitochondrial toxicity leading to cholestasis and hepatocellular injury (Fattinger et al. 2001 and Kenna et al. 2015), indicating spheroid models may be effective tools to identify the contribution of bile-acid transport inhibition in hepatocellular injury. Multiplexed in vitro assays, including 3D models in addition to physicochemical descriptors could lead to the most encompassing DILI strategy as it builds on many of the strategies outlined above (e.g. Williams et al. 2019).

**Table 2:**
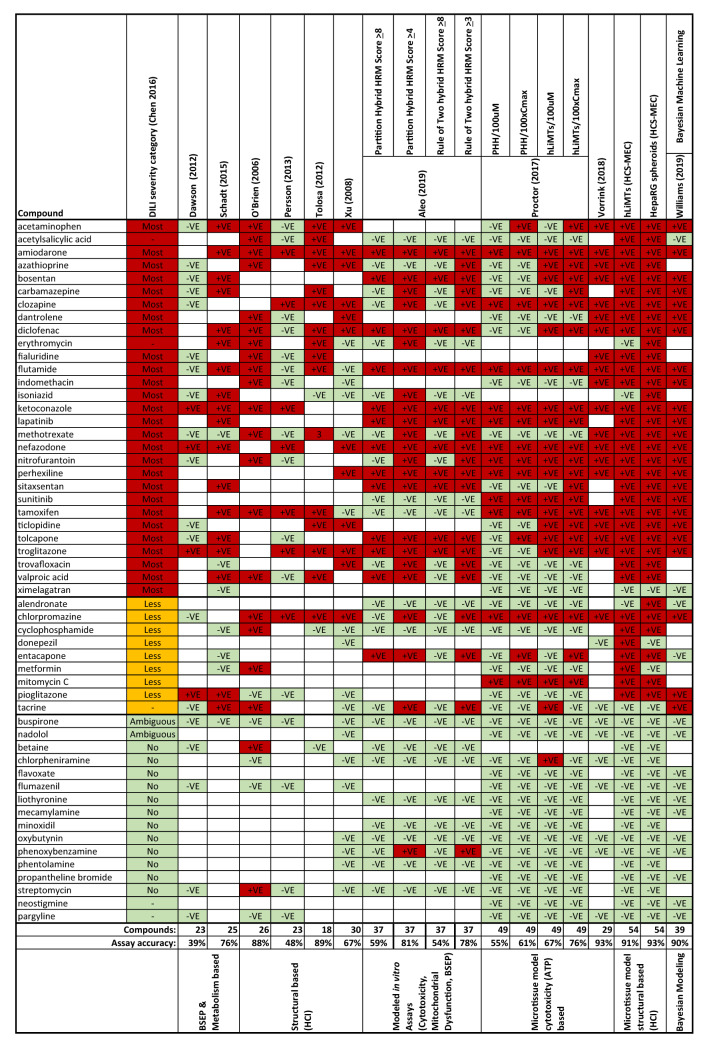
Distribution of drug-induced liver injury (DILI) in vitro assay prediction across 8 literature references using either hLiMTs or HepaRG spheroids normalised against Chen et al. (2016) DILI severity category (color table online)

A multiplexed approach appears the optimal way to detect multiple DILI mechanisms. An approach such as HCI potentially allows for the detection of multiple DILI mechanisms within a single cell (e.g. Saito et al. 2016). HCI offers the ability to detect cellular pathways associated with sub-lethal toxicities allowing a sensitive early indication. Many conventional cytotoxicity assays (e.g. LDH release, MTT, cellular ATP) may well be less sensitive as these endpoints are typically cell health markers. The combination of multiple endpoints not only allows for a much better understanding of the mechanisms involved, but, with well-chosen drug exposure times (and potentially the use of live cell imaging), it also permits an insight into the sequence of events and mechanisms leading to toxicity. This is not always possible with conventional cytotoxicity assays, most of which are terminal in nature. In addition, the possibility to measure multiple endpoints simultaneously with live HCI using cellular dyes in a high-throughput manner could dramatically decrease the number of assays, experiment time and therefore experimental cost. Indeed, HCI approaches have been used to profile cells morphologically (Cell Painting), whereby 8 cellular components or organelles are imaged simultaneously, measuring size, shape, texture, intensity, etc. leading to over 1500 morphological features (Bray et al. 2016); however, this approach has not yet been applied in DILI strategies.

Many pharmaceutical strategies reviewed here describe the development of mechanistic multi-endpoint-based assays in 2D cellular models. To date, only limited studies have combined multi-parametric analysis combined with 3D approaches (e.g. Williams et al. 2019). Often only biochemical endpoints such as ATP have been adopted. As a proof of concept Proctor et al. (2017) evaluated dose- and time-dependent release of α-GST, total levels of HMGB1, and relative expression of miR-122 in the supernatants of individual spheroids; however, responses were observed to coincide with ATP content. In our laboratories, a combined HCI 3D approach has been developed, which allows the simultaneous assessment of multiple markers of cell health alongside a terminal measure of cellular ATP following chronic compound exposure. 3D spheroid models consisting of either primary human hepatocytes with non-parenchymal cells (hLiMT’s) or HepaRG cells (HepaRG spheroids) were assessed for size (MS), oxidative stress (OS), MMP, mitochondrial mass (MM), GSH and cellular ATP content following drug exposure. Fluorescent images are acquired using the confocal mode of an ArrayScan™ XTI HCI reader (ThermoScientific) following which cellular ATP was measured using 3D CellTiter-Glo (Promega). One of the disadvantages levied at this type of approach is its relative expense (Proctor et al. 2017). However, this approach can be performed using automated 96- or 384-well plate formats making it amenable to early-stage screening assessment.

**Fig. 2:**
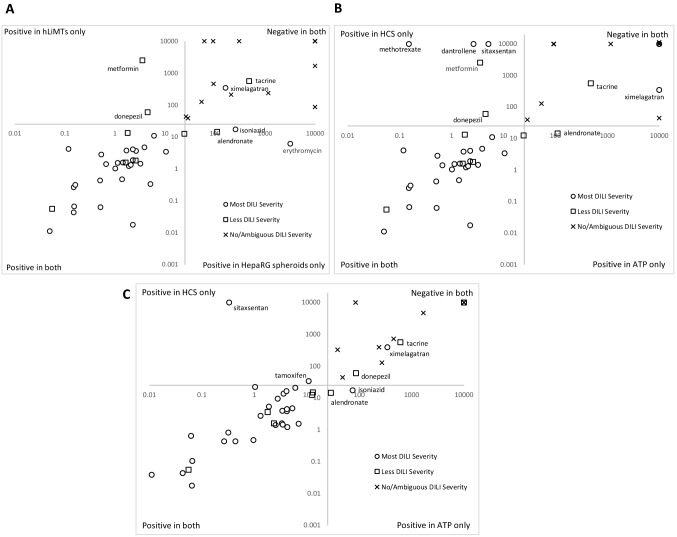
**a** Global comparison of therapeutic index (TI) for hLiMT’s with hepaRG spheroids for 54 compounds, assigned DILI severity categories taken from Chen et al. (2016) when available otherwise average literature category used. HepaRG spheroids plotted on the *y*-axis and hLiMT’s plotted on the *x*-axis. **b** Comparison of therapeutic index (TI) for high-content screening (HCS) endpoints alone with cellular ATP alone in hLiMTs **c** Comparison of therapeutic index (TI) for high-content screening (HCS) endpoints alone with cellular ATP alone in HepaRG spheroids. Open circle, most DILI severity; open square, less DILI severity; cross, no/ambiguous DILI severity. Axis crossing set at 25 to represent a 25 × *C*_max.tot_ cut-off. MEC < 25 × *C*_max.tot_ cut-off applied for DILI severity categories (color figure online)

The National Toxicology Program (NTP) of National Institutes of Health (NIH) in 2017 showed that HepaRG spheroids exhibit physiologically relevant levels of xenobiotic metabolism (CYP1A2, CYP2B6, and CYP3A4/5) using probe substrate activity assays and maintained a stable phenotype up to at least 28 days in culture (Ramaiahgari et al. 2017). HepaRG 3D spheroids offer an alternative to primary human hepatocytes models which have limited cell batches and may exhibit donor variability as previously discussed. As we have seen hLiMTs display slightly lower sensitivity than HepaRG spheroids (87% vs 89% respectively), with high specificity (100% in both). A comparison of hLiMTs against HepaRG spheroids as shown in Fig. 1 highlights a good correlation between the models. A subset of compounds, however, respond differentially, e.g. metformin and donepezil, both less severe DILI compounds that are predicted as positive for DILI potential in hLiMTs but not HepaRG spheroids. Isoniazid and erythromycin, less severe DILI compounds, and alendronate, a most severe DILI compound, were positive in HepaRG spheroids alone, using the 25 × *C*_max.tot_ cut-off. Given the complexity of DILI, it appears multi-parametric assays combined with repeated dosing regimens should improve cost-efficiency in early-stage screening and also sensitivity to DILI mechanisms, including reactive metabolites, mitochondrial perturbations and cholestatic liabilities. If HCI is compared with the predictive strength of ATP alone (Fig. 2) for hLiMTs and HepaRG spheroids, an overall good correlation is observed. In the hLiMTs assay, however, HCI alone allows the detection of the in vivo positive DILI compounds methotrexate, dantrolene, donepezil, metformin and sitaxsentan which go unpredicted with ATP alone, whereas HepaRG spheroids’ HCI alone determines sitaxsentan and tamoxifen as positive. Many of these compounds were also shown to be negative in hLiMTs with an ATP endpoint (Proctor et al. 2017).

## Inter-lab variability in applied human exposure estimates

As discussed several DILI strategies use *C*_max.tot_ normalisation of in vitro data to improve in vivo extrapolation; however, there is a high degree of variability of plasma *C*_max.tot_ values used. Figure 1 displays the distribution of *C*_max.tot_ values and DILI categorisation for 45 compounds across 9 literature references where in vivo exposure has been used for normalisation. Flutamide, for example, shows ambiguous hepatotoxicity prediction across the literature, with a 16.6-fold difference in *C*_max.tot_ values utilised across studies (0.36 µM to 6 µM). Xu et al. (2008) predicted flutamide as negative for hepatotoxicity using normalisation to a *C*_max.tot_ value of 0.36 µM, whereas O’Brien et al. (2006) predicted positive hepatotoxicity utilising higher *C*_max.tot_ values of 6 µM. For data analysis, the median *C*_max.tot_ from the 9 literature references displayed in Fig. 1 was utilised in an attempt to minimise ambiguity often found across literature data normalisation. To align closely in vitro analysis with Vorrink et al. (2018), a cut-off of IC_20_ for the first responding assay endpoint was used and normalised to total average plasma *C*_max.tot_, to define the lowest therapeutic index (TI) (Table 3). Using an MEC < 25 × *C*_max.tot_ cut-off, this approach resulted in sensitivities of 87% and 89% for hLiMTs and HepaRG spheroids, respectively, with specificities of 100% (Table 4; accuracy 91% and 93%, respectively), confirming the high specificity observed using 3D approaches (HCI methodology and data analysis previously described; Longo et al. 2019).

**Table 3:** Assigned DILI category (Chen et al. 2016 or literature alignment), plasma *C*_max.tot_ values, *f*_u_ values, MEC and AC_50_ values for hLiMT’s and HepaRG spheroids in the high-content screening (HCS) and cellular ATP endpoints

						hLiMT's						hepaRG spheroid					
Compound	DILI severity category (Chen et al. 2016)	Assigned DILI category	Human plasma *C*_max_ (µM)	*f*_u_	Human plasma *C*_max,u_ (µM)	HCS endpoint MEC (µM)	First responding feature	HCS endpoint AC50 (µM)	First responding feature	ATP endpoint MEC (µM)	ATP endpoint AC50 (µM)	HCS endpoint MEC (µM)	First responding feature	HCS endpoint AC50 (µM)	First responding feature	ATP endpoint MEC (µM)	ATP endpoint AC50 (µM)
Acetaminophen	Most	3	152.000	0.920	139.840	456	MS	4340	GSH	292	1240	656	MS	3100	GSH	187	760
Amiodarone	Most	3	4.450	0.020	0.089	6.51	OS	28.2	MMP	4.58	5.33	4.62	MS	13.1	GSH	98.8	112
Azathioprine	Most	3	7.212	0.700	5.048	1.58	MS	41.6	GSH	0.361	1.18	0.0805	MMP	2	GSH	0.278	0.48
Bosentan	Most	3	7.430	0.020	0.149	63	MMP	179	MMP	29.4	57.9	40	MMP	125	MMP	35.2	74.7
Carbamazepine	Most	3	50.790	0.240	12.190	109	GSH	226	GSH	73.6	145	172	GSH	343	GSH	81.5	159
Clozapine	Most	3	2.448	0.030	0.073	5.61	GSH	15.2	MM	10.5	14	4.66	MS	13.2	MS	13.1	16.6
Dantrolene	Most	3	3.946	0.140	0.552	10.5	MMP	51	GSH	NR	NR	14.5	MM	174	GSH	53.2	100
Diclofenac	Most	3	10.078	0.010	0.101	45	OS	293	GSH	17.2	62.1	42.9	GSH	98.6	GSH	38.2	78
Fialuridine	Most	3	0.820	0.360	0.295	6.54	MS	NR	NR	0.932	6.3	5.78	GSH	115	GSH	1.28	9.16
Flutamide	Most	3	5.431	0.050	0.272	3.63	OS	43.5	GSH	18.5	29.9	13.9	MS	42.7	MMP	7.75	14.9
Indomethacin	Most	3	8.380	0.010	0.084	95.2	MMP	672	MMP	4.54	9.29	23.8	OS	215	GSH	79.2	94.2
Isoniazid	Most	3	76.564	0.970	74.267	NR	NR	NR	NR	NR	NR	5790	OS	14,000	GSH	1330	3520
Ketoconazole	Most	3	11.290	0.050	0.565	5.88	GSH	12.4	GSH	6.84	9.09	0.713	OS	15.4	GSH	7.27	8.42
Lapatinib	Most	3	11.610	0.010	0.116	1.79	OS	30.7	MMP	12.6	30.1	0.768	GSH	2.25	OS	1.21	2.11
Methotrexate	Most	3	4.630	0.500	2.315	0.703	GSH	16.9	GSH	NR	NR	0.2	GSH	0.2	GSH	0.2	0.2
Nefazodone	Most	3	4.255	0.010	0.043	13.8	GSH	23.4	GSH	8.98	12.1	5.67	MS	23.8	GSH	11.6	19.4
Nitrofurantoin	Most	3	20.994	0.310	6.508	19.4	MS	73.6	GSH	3.21	11.7	5.59	MMP	17.5	GSH	9.27	11.6
PERHEXILINE	Most	3	2.162	0.100	0.216	0.354	MMP	1.75	GSH	1.49	1.69	0.693	MS	6.32	GSH	1.76	3.69
Sitaxsentan	Most	3	25.289	0.005	0.126	130	MMP	NR	NR	NR	NR	8.53	MMP	9.74	MMP	NR	NR
Sunitinib	Most	3	0.068	0.050	0.003	0.157	GSH	0.421	GSH	0.347	0.4	0.281	GSH	1.71	GSH	1.1	3.11
Tamoxifen	Most	3	0.320	0.010	0.003	1.97	OS	7.49	GSH	3.93	6.13	3.52	MS	16.7	GSH	10.8	12.7
Ticlopidine	Most	3	8.075	0.020	0.161	22.7	OS	115	GSH	0.957	10.7	34	MM	60.8	MM	36.2	56
Tolcapone	Most	3	47.577	0.001	0.048	24.1	GSH	60.8	GSH	114	161	21	GSH	36.1	GSH	20.5	35.3
Troglitazone	Most	3	6.387	0.010	0.064	67.6	MM	73.6	MMP	74	89.5	22	MMP	33	GSH	25	28.8
Trovafloxacin	Most	3	5.020	0.240	1.205	20.1	MM	38.9	GSH	16.4	29.4	17.5	GSH	24.9	GSH	7.27	12.9
Valproic acid	Most	3	693.426	0.080	55.474	1600	GSH	NR	NR	6220	7080	45.6	MS	148	GSH	12.3	41.3
Ximelagatran	Most	3	0.380	0.210	0.080	NR	NR	NR	NR	61.8	NR	133	MS	NR	NR	149	NR
Acetylsalicylic acid	–	3	1380.062	0.390	538.224	1930	GSH	4690	GSH	2270	3250	1340	MS	NR	NR	659	1630
Erythromycin	–	3	6.198	0.100	0.620	NR	NR	NR	NR	NR	NR	38.1	MS	294	GSH	128	214
Alendronate	Less	2	0.020	0.220	0.004	2.24	OS	15.6	GSH	2.86	7.71	0.58	GSH	3.13	GSH	0.291	0.822
Chlorpromazine	Less	2	0.941	0.050	0.047	2.47	MM	6.41	GSH	2.7	3.73	1.72	MS	8.8	GSH	3.45	6.33
Cyclophosphamide	Less	2	143.000	0.800	114.400	236	GSH	992	GSH	613	906	340	MS	1860	GSH	229	419
Donepezil	Less	2	0.107	0.040	0.004	0.487	MMP	7.19	MMP	3.17	9.76	9.43	MM	NR	NR	6.49	NR
Entacapone	Less	2	3.588	0.020	0.072	88.8	GSH	139	GSH	125	150	45.4	GSH	129	MM	45.5	115
Metformin	Less	2	7.821	0.990	7.743	28.1	MS	NR	NR	503	768	NR	NR	NR	NR	NR	NR
Mitomycin C	Less	2	7.100	0.760	5.396	0.399	OS	1.09	GSH	0.454	0.929	0.4	GSH	0.4	GSH	0.4	0.4
Pioglitazone	Less	2	2.946	0.010	0.029	5.35	GSH	9.95	MMP	38.1	106	39.2	GSH	93.7	MMP	44.7	67.5
Tacrine	–	2	0.080	0.450	0.036	38.5	MM	52.4	GSH	44.7	41.8	48.7	GSH	65.6	GSH	45.3	51.8
Betaine	No	1	944.008	0.760	717.446	NR	NR	NR	NR	NR	NR	NR	NR	NR	NR	NR	NR
chlorpheniramine	No	1	0.366	0.277	0.101	66.6	GSH	98.5	GSH	10.7	30.9	14.3	MS	119	MM	120	129
Flavoxate	No	1	1.788	NA	NA	NR	NR	NR	NR	111	127	NR	NR	NR	NR	NR	NR
Flumazenil	No	1	1.121	0.580	0.650	NR	NR	NR	NR	NR	NR	NR	NR	NR	NR	NR	NR
Liothyronine	No	1	0.002	0.020	0.00005	NR	NR	NR	NR	NR	NR	4.14	SIZE	82.8	MMP	11.5	30.5
Mecamylamine	No	1	0.142	0.600	0.085	NR	NR	NR	NR	NR	NR	12.2	MMP	NR	NR	NR	NR
Minoxidil	No	1	1.195	0.990	1.183	111	MMP	NR	NR	NR	NR	NR	NR	NR	NR	NR	NR
Oxybutynin	No	1	0.022	0.030	0.001	2.11	GSH	6.06	GSH	NR	NR	10.3	MM	64.5	GSH	16.1	28.5
Phenoxybenzamine	No	1	0.197	0.009	0.002	15.6	GSH	NR	NR	10.7	26.9	53.8	MS	NR	NR	25.3	NR
Phentolamine	No	1	0.075	0.460	0.034	87.4	SIZE	NR	NR	NR	NR	18.1	GSH	63	MM	29.3	35.2
Propantheline bromide	No	1	0.440	NA	NA	NR	NR	NR	NR	NR	NR	NR	NR	NR	NR	NR	NR
Streptomycin	No	1	64.481	0.650	41.912	NR	NR	NR	NR	1700	1960	3140	GSH	NR	NR	2830	3650
Buspirone	Ambiguous	1	0.010	0.140	0.001	NR	NR	NR	NR	3.12	5.15	NR	NR	NR	NR	NR	NR
Nadolol	Ambiguous	1	0.420	0.700	0.294	NR	NR	NR	NR	NR	NR	NR	NR	NR	NR	NR	NR
Neostigmine	–	1	0.045	0.800	0.036	NR	NR	NR	NR	NR	NR	NR	NR	NR	NR	NR	NR
Pargyline	–	1	0.819	0.290	0.237	NR	NR	NR	NR	NR	NR	NR	NR	NR	NR	NR	NR

**Table 4:** Sensitivities, specificities and accuracies in hLiMTs and HepaRG spheroids either as a combined assay (HCS and ATP endpoints), HCS or ATP alone normalised to either 25 × *C*_max.tot_ or 100 × *C*_max,u_

	Combined assay (MEC/25 × *C*_max_)	ATP only (MEC/25 × *C*_max_)	HCS only (MEC/25 × *C*_max_)	Combined assay (MEC/100 × *C*_max_,u)	ATP only (MEC/100 × *C*_max_,u)	HCS only (MEC/100 × *C*_max_,u)
hLiMTs						
Sensitivity	87%	71%	87%	61%	45%	55%
Specificity	100%	100%	100%	86%	93%	93%
Accuracy	91%	80%	91%	67%	58%	65%
HepaRG spheroids						
Sensitivity	89%	84%	84%	63%	50%	61%
Specificity	100%	100%	100%	93%	93%	93%
Accuracy	93%	89%	89%	71%	62%	69%

**Fig. 3:**
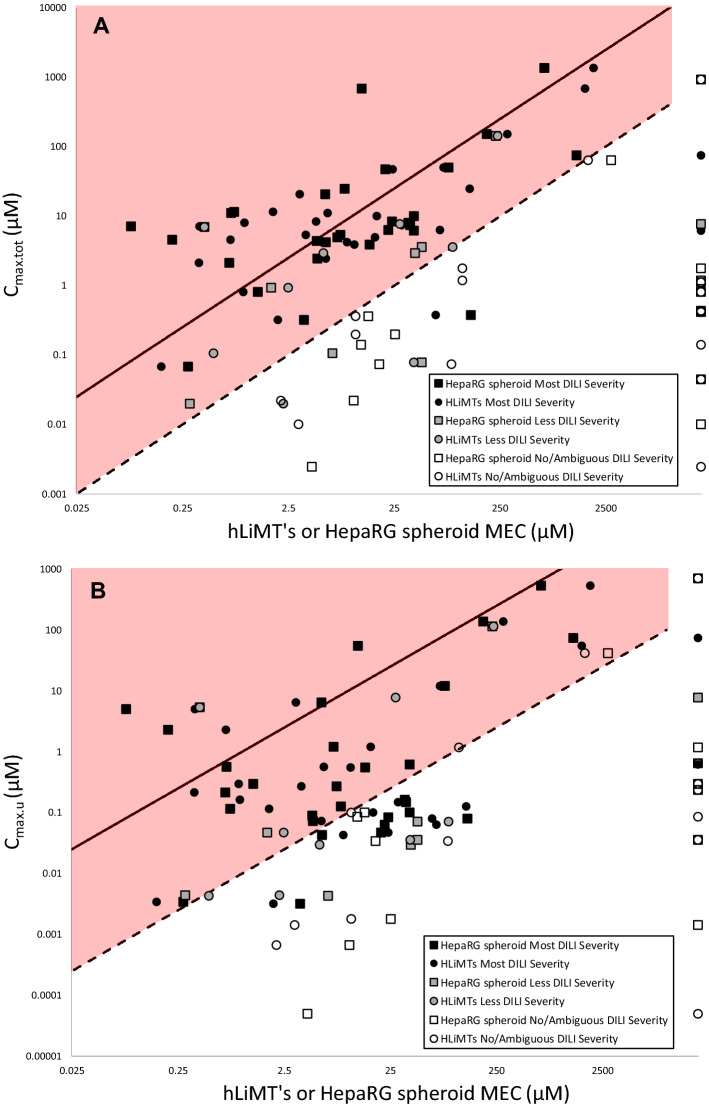
Correlation of hLiMT and hepaRG spheroid minimal effective concentration (MEC) of the first responding feature (µM) with either **a** plasma *C*_max.tot_ (µM) or **b** *C*_max,u_ (µM). Assigned DILI potential categories are taken from Chen et al. (2016) when available otherwise average literature category used. Squares are hepaRG spheroids and circles are hLiMTs, severe DILI potential; closed squares or circles, less DILI potential; grey squares or circles and no/ambiguous DILI potential; open squares or circles. Dashed line represents an MEC < 25 × *C*_max.tot_ or 100 × *C*_max,u_ cut-off. Solid black line represents 1 × *C*_max.tot_ or *C*_max,u_. Red shading highlights area of positive DILI potential. Non-responding compounds assigned an arbitrary value of 20,000 µM (color figure online)

One current limitation of the methodologies used to translate in vitro assay data is the continued use of *C*_max.tot_. As discussed *C*_max.tot_ values are routinely used for data normalisation during validation of early-stage screening strategies incorporating the bound and unbound drug fractions in the clinic. However, it is becoming more widely accepted that this approach may lead to misprediction of potential DILI compounds. Drugs with promising therapeutic potential may have a high total plasma concentration but low plasma-free fraction (*f*_u_), therefore maybe less likely to ultimately cause DILI in the clinic. This situation is reminiscent of the journey for quantitative drug–drug interaction (DDI) analyses. After many years, several laboratories promoted the scientific basis for consideration of unbound exposures (systemic and/or liver) in such evaluations (see Riley and Wilson 2015; Williamson and Riley 2017). Whilst the application of total exposures protected patients since it minimises false-negatives, the potential exists to delay or even terminate the progress of innovative therapies. The most recent regulatory draft guidance have recognised the progress in this area and now include algorithms incorporating unbound exposure (US Food and Drug Administration 2017). Investigative DILI scientists may well benefit from a re-familiarisation of this area given the obvious corollaries. The prediction of DILI severity was evaluated further using the 3D combined HCI assays using plasma-free fraction. The majority of *f*_u_ values were obtained from literature, when unavailable in-house prediction was utilised. Unbound plasma *C*_max_ concentrations (*C*_max,u_) were derived by multiplying total plasma *C*_max_ with the corresponding free fraction (*f*_u_) for each compound. Normalising the in vitro data to 25 × *C*_max,u_ reduced the overall sensitivity of both hLiMT and HepaRG spheroids to 45% and 40%, respectively, with no change in specificity (100% in both models; Accuracy 60% and 66%, respectively). Increasing the *C*_max,u_ cut-off to 100 × increased the sensitivity slightly to 61% and 63%, respectively, however with a compromise on specificity falling to 86% and 93%, respectively (Table 4 and Fig. 3; increasing assay accuracy to 67% and 71%, respectively). Similar findings have been shown in previous studies, with reduced sensitivity observed with unbound concentration normalisation (e.g. Shah et al. 2015, Albrecht et al. 2019). However, as with DDIs, analyses using unbound exposure and in vitro cytotoxicity revealed compounds with additional, contributory mechanisms (e.g. ximelagatran) and known substrates for hepatic uptake transporters such as OATP1B1 (bosentan, sitaxsentan) and OAT2 (diclofenac, indomethacin, tolcapone). It is likely that the hepatic exposure for the latter subset of compounds is under-estimated from plasma estimates and a correction is needed to adjust the in vitro risk as demonstrated for CYP DDIs (Grime et al. 2008; Treyer et al 2019). An alternative to normalising in vitro DILI data to *C*_max_ would be to link efficacious oral doses and associated blood concentrations of test compounds to the in vitro probability of hepatotoxicity, as recently proposed by the Horizon 2020 EuToxRisk project (Albrecht et al. 2019).

## Future perspectives

Further complex endpoints, which can resolve subtle alterations in cellular health or indeed the molecular initiating events (MIEs), may enhance DILI prediction using 3D models. Indeed, gene expression alterations specific for cholestasis, steatosis, or genotoxicity have been identified in PHH spheroids (Bell et al. 2017). Recently, the National Toxicology Program (NTP) have further utilised HepaRG cells and combined with high-throughput transcriptomics (HTT; TempO-Seq) to evaluate 24 compounds. A positive was considered if ≥ 105 respective benchmark concentrations (BMCs) was observed in relation to 10 × human *C*_max.tot_. This approach identified six compounds associated with DILI, several of which have also been identified by PHH spheroids (troglitazone, acetaminophen, trovolfoxacin and rifampicin; Vorrink et al. 2018). Even though a larger reference set of compounds is warranted, linking gene signatures and pathway analysis with mechanism of action will hopefully add further mechanistic understanding aiding DILI prediction and risk assessment especially if combined with 3D human hepatic models. Interestingly, one of the compounds that is consistently not picked up in any of the reviewed DILI strategies is the idiosyncratic DILI (iDILI) drug ximelagatran. The thrombin inhibitor was removed from the market following altered hepatic function in 7.9% of patients, with pharmacogenomics data indicating immunogenic pathogenesis (Keisu and Andersson 2010). Idiosyncratic responses such as these are difficult to recapitulate in vitro; however, monocyte-derived hepatocyte-like cells (MH cells), derived from patients diagnosed with iDILI, have showed concordance with in vivo (Benesic et al. 2019). As MH cells, however, are limited in production volumes it is difficult to envision how these cells could be applied routinely in early DILI screening strategies.

A natural evolution of 3D cellular models has been in the development of microphysiological cell-based systems which are, in simplistic terms, microfluidic devices designed to support an in vitro physiological environment that replicates human biology in vivo. As with the development of 3D models from 2D systems the drive in this field has been to replicate more closely the structure and biology of that in human and so aim to improve translatability of in vitro models to the human responses. The use of perfused in vitro systems mimics the micro-environmental factors of the intact liver such as haemodynamics and sheer stress, both of which have been shown to improve hepatocyte functionality, metabolic activity and morphology (Dash et. al. 2013). In addition, a non-circulatory approach could allow for the clearance of metabolised products as well as generating microenvironmental biomolecular gradients.

This emergence of microphysiological systems (MPS) as a potential model for the pharmaceutical industry is reflected in the number of recent publications and industry perspectives in the field (Ewart et al. 2017, 2018). This has also been expressed in the increasing number of commercial companies in this field such as TissUse and Emulate, all of whom are focused on developing MPS. The approaches have focused on two types of systems: liver-on-a-chip devices and multi-organ linked MPS. For example, the National Centre for Advancing Translational Sciences (NCATS) has a major initiative termed ‘Tissue Chip’ which aims to develop organ-on-a-chip models for every major organ of the human body including diseased versions. Several NCATS supported teams are working on the liver-on-a-chip model, in particular, a team at the University of Pittsburgh have created a liver-on-a-chip model utilising fluorescent biosensor cells to relay visually changes in cellular function such as death or free radical damage following drug exposure. A recent publication by Ma et al. (2018) describes an alternative liver-on-a-chip approach whereby a 3D hepatic spheroid in situ perfusion model has been designed and fabricated. They describe the development of a biomimetic microenvironment which permits the high-throughput parallel perfusion of 1080 HepG2/C3A spheroids using a concave microwell-based-PDMS-membrane multilayer chip. Utilising this model, they found improved longevity, cellular polarisation, liver-specific functions and metabolic activity of the spheroids suggesting a closer correlation with in vivo cellular physiology than observed with traditional 2D methods. Examples of organ-linked approaches include systems where an integrated liver-kidney chip have been developed which have demonstrated characterisation of drug metabolism and assessment of subsequent nephrotoxicity (Li et al. 2018). In addition, functional coupling of multiple organ systems has been developed. Vernetti et al. (2017) recently reported on a multi-organ system representing the major absorption, metabolism and clearance organs along with skeletal and neurovascular models. They demonstrated organ-specific processing of three reference compounds whose data were consistent with clinical observations. Even though liver on a chip and multi-organ devices are still in the early phase of development, they do show promise in improving prediction of toxicity and show great applicability for use in preclinical drug development (Starokozhko and Groothius 2017). To date a comprehensive DILI reference set of compounds has not been evaluated using an MPS system; therefore, the evaluation against current pharma DILI strategies is not yet possible. AstraZeneca evaluated the use of a human liver-chip model versus a PHH liver spheroid model for acetaminophen (APAP) and fialuridine (FIAU). Similar to the finding reported here both “Most-DILI” compounds were detected in 3D microtissues and furthermore, both the liver-chip model and microtissue exhibited comparable sensitivity (Foster et al. 2019).

All of the current published pharmaceutical company strategies to date have focused on NCEs, therefore, as the industry progresses with diversification into the development of biological and non-biological modalities, the applicability of these in vitro tools in safety assessment will need to be addressed, with emerging gaps filled.

## Summary and conclusions

This review has focused on assessing the pharmaceutical led DILI strategies over recent years. This has highlighted challenges due to variable DILI classification; lack of overlapping reference compound sets; diversity in test systems and assay formats, including duration of exposure; differences in assignment of significance effects in assays; varying approaches to exposure normalisation. FDA initiatives such as the DILIrank dataset (Chen et al 2016) should aid standardisation. With this in mind, 3D liver models are showing early promise with overall improved DILI prediction accuracy, and combination with multi-parametric endpoint analysis such as HCI or even high-throughput transcriptomics will improve mechanistic understanding and potential sensitivity to non-cytotoxic DILI mechanisms. Emerging trends in DILI strategies are starting to occur, with multi-mechanistic assays, combined with organotypic 3D models and physiochemical descriptors, normalised to in vivo exposure. To improve extrapolation and translation to clinical DILI risk analysis it is important to contextualise these parameters with human (unbound) exposure. As the experience in the DDI arena has demonstrated this should provide confidence and ultimately acceptance by Regulatory authorities (US Food and Drug Administration 2017). The latter should evolve to encompass improved estimates of liver exposure accounting for altered intra-cellular concentrations due to drug transporters as established in the DDI field. Finally, modelling or Bayesian machine learning approaches should move us beyond binary endpoint analysis aiding our compound selection in drug discovery.

## Electronic supplementary material

Below is the link to the electronic supplementary material.

Supplementary file1 (XLSX 305 kb)

Supplementary file2 (XLSX 17 kb)
